# Neonatal Intestinal Obstruction: When to Suspect Duplication Cyst of Bowel as the Cause

**DOI:** 10.21699/jns.v5i4.467

**Published:** 2016-10-10

**Authors:** Rizwan Ahmad Khan, Shagufta Wahab, Imran Ghani

**Affiliations:** 1 Department of Pediatric Surgery, J. N. Medical College Hospital, A.M.U. Aligarh; 2 Department of Radiodiagnosis, J. N. Medical College Hospital, A.M.U. Aligarh

**Keywords:** Intestinal obstruction, Duplication cyst, Neonate

## Abstract

Background: Duplication cyst is a rare cause of neonatal intestinal obstruction. Their most common location is the small intestine. The clinical presentation is extremely variable depending upon its size, location and type and the age of the patient and are mainly encountered during infancy or early childhood. The diagnosis is very difficult in neonates. This study was undertaken to study their presentation, diagnostic modality of choice and further management in neonatal age group.

Materials and Methods: This was a retrospective study performed at the Department of Paediatric Surgery, J .N Medical College Hospital, AMU Aligarh from July 2008 to June 2014. The data was analyzed with respect to demographic profile of the neonates, their initial clinical presentation, radiological features and subsequent event leading to intervention, operative features and outcome.

Results: There were a total of seven neonates between ages of 3 days and 21 days who were diagnosed as cases of intestinal obstruction due to duplication cyst. The majority of the patients were having ileal duplication cyst (n=4). Ultrasonography played important role in majority of the cases for diagnosis. There was one patient in which the diagnosis was confused with ileal atresia. All the patients underwent excision with restoration of bowel continuity.

Conclusion: The diagnosis of intestinal obstruction in neonate due to duplication cyst is difficult. It has varied presentation and preoperative diagnosis at times may be challenging. Surgery is the mainstay of the treatment.

## INTRODUCTION

Duplication cysts are rare congenital anomalies of gastrointestinal tract (GIT) causing various symptoms which are nonspecific leading to difficulty in management. They are most commonly observed in the small bowel but they can be present in any part of GIT. It may present at any age but the most common age is childhood. The most common presentation is intestinal obstruction. It may also present as perforation and gastrointestinal bleeding which are mostly seen as its complications. We have done a retrospective study about duplications cysts presented with neonatal intestinal obstruction.

## MATERIALS AND METHODS

We performed a retrospective analysis of the data available in the unit of Paediatric Surgery, J.N. Medical College Hospital, AMU, India. We studied the medical record file of the babies who presented as intestinal obstruction in neonatal period from July 2008 to June 2014. Out of these patients we found seven cases of neonatal obstruction whose final diagnosis was duplication cyst. In these patients we studied the demographic profile, initial clinical presentation, complication or event leading to surgery, radiological investigations, operative findings and outcome.

## RESULTS

Demography: There were a total of seven cases of neonates who were diagnosed as cases of intestinal obstruction due to duplication cyst or its complications during the study period. There was male preponderance and the male female ratio was 5:2. Most of the babies presented during first week of life (n=4). Table 1 depicts the demographic profile and other parameters studied in the patients. 

**Figure:**
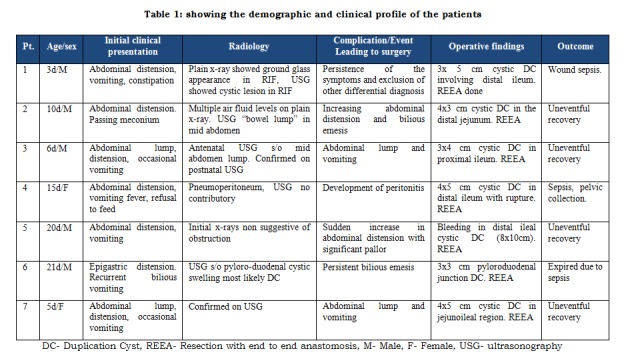
Table 1: showing the demographic and clinical profile of the patients

Presentation: The most common presentation was abdominal distension associated with vomiting. It was noted that three patients had complaints suggestive of incomplete obstruction. One patient (Patient no.5) presented with incomplete obstruction which on conservative treatment showed improvement on first day but later on developed bleeding inside the cyst which became evident due to increasing abdominal distension and associated pallor demanding urgent surgical exploration. 

Investigations: All the patients underwent routine biochemical and specific investigations. Plain X-ray abdomen was done in all the patients as preliminary investigation. This was suggestive of multiple air-fluid level in all except two patients. Ultrasonography was suggestive of some cystic pathology in all except one patient. There was one patient in whom antenatal diagnosis was suggested by ultrasonography. 

Management: All the babies underwent exploratory laparotomy with cyst excision and restoration of bowel continuity by end to end anastomosis. In four patients bowel compression leading to obstruction was the reason for laparotomy. While one patient each had volvulus, intracystic bleed and perforation as the reason for exploration. The most common region affected was ileum (n=4). There was one patient each of pyloro-duodenal and jejunal duplication cyst (Table 1). In all seven cases it was the cystic variant which was found and measured from 3 to 10 cm in size. All cysts were lined by normal bowel mucosa except one which has additionally ectopic gastric tissue in it. 

Outcome: One patient died in the postoperative period because of septicemia. This patient had pyloro-duodenal duplication cyst. Four patients had uneventful recovery while two patients had delayed recovery due to wound and abdominal sepsis.

Follow up: All six patients were healthy in their first follow up visit within 4 weeks of discharge. One patient (Patient no.4) had poor weight gain. Overall follow-up is of 1 year except in one patient (6months follow-up).

## DISCUSSION

The duplication cyst was first reported by R H Fitz in 1884. This was later described by other authors as well [1]. The most important criterion for the diagnosis of duplication cyst is the presence of normal gastrointestinal epithelial lining in it. Other criteria include the presence of surrounding smooth muscle and continuity with the alimentary tract. In our series all criteria are met.

Duplication cyst can be found anywhere in the alimentary tract from the mouth to the anus. They are most commonly seen in the ileal region of the small bowel as was observed by Puligandla et al [2]. In our study also ileum was the most common site. Pyloro-duodenal, colonic and rectal cysts are rare. They can be cystic or tubular in shape and always lie on the mesenteric side of the bowel. Although they can cause symptoms at any point in life, they usually present during infancy. They cause symptoms depending upon their size, shape, location and presence of complications. The complications include bleeding into the cyst, intestinal obstruction, intestinal or duplication cyst perforation, volvulus, cyst torsion, cystic rupture, and malignancy (sarcoma, lymphangiosarcoma) [3].

The diagnosis of duplication cyst in the neonatal period is extremely difficult because the symptomatology is nonspecific. Partial intestinal obstruction in a neonate is an important symptom that should let the suspicion of the entity. The diagnosis is mainly by excluding other more common causes of neonatal intestinal obstruction with radiological diagnostic aid. Ultrasound is the most widely used and the first imaging modality used in the investigation of suspected cases of duplication cysts. CT and MRI scans can give better anatomic localization. On USG, the diagnosis is suggested by the presence of a hypoechoic outer muscular layer and echogenic internal mucosal layer [4]. Peristaltic muscular contractions of the cyst wall are highly suggestive of an intestinal origin of the cyst. Antenatal diagnosis of duplication cyst is suggested by some authors but it is difficult [5]. In our study one patient presented with antenatal USG which was suggestive of lump in mid abdomen.

There are other differential diagnoses which should be considered in neonates presenting with abdominal lump with features of incomplete obstruction. These include ovarian cysts, mesenteric and omental cysts [6]. However in neonates, the main differential diagnosis that needs to be ruled out is intestinal atresia which sometimes leads to such gross proximal bowel dilatation which may mimic cystic lesion on USG leading to diagnostic dilemma as happened in the patient no 1 in our study.

The treatment of choice is surgical excision with restoration of bowel continuity. This is easily achieved in cystic variants. However in tubular duplication cysts there is involvement of large portion of bowel which on resection might result in short bowel syndrome. Therefore in these cases mucosal stripping is suggested thereby preventing risk of peptic ulceration or carcinogenesis [7]. In our series, all cases had cystic variety and excision with bowel continuity restoration was achieved uneventfully.

## CONCLUSION

Duplication cysts are rare cause of neonatal intestinal obstruction. Diagnosis can be achieved keeping a high index of suspicion in a neonate with symptoms suggestive of incomplete or complete bowel obstruction and sonographic evidence of an intra-abdominal cystic swelling located in close proximity to mesenteric border. Management is straightforward surgical with good outcome.

## Footnotes

**Source of Support:** None

**Conflict of Interest:** None
